# Outbreak analytics: a developing data science for informing the response to emerging pathogens

**DOI:** 10.1098/rstb.2018.0276

**Published:** 2019-05-20

**Authors:** Jonathan A. Polonsky, Amrish Baidjoe, Zhian N. Kamvar, Anne Cori, Kara Durski, W. John Edmunds, Rosalind M. Eggo, Sebastian Funk, Laurent Kaiser, Patrick Keating, Olivier le Polain de Waroux, Michael Marks, Paula Moraga, Oliver Morgan, Pierre Nouvellet, Ruwan Ratnayake, Chrissy H. Roberts, Jimmy Whitworth, Thibaut Jombart

**Affiliations:** 1 Department of Health Emergency Information and Risk Assessment, World Health Organization, Avenue Appia 20, 1211 Geneva, Switzerland; 2 Department of Infectious Hazard Management, World Health Organization, Avenue Appia 20, 1211 Geneva, Switzerland; 3 Faculty of Medicine, University of Geneva, 1 rue Michel-Servet, 1211 Geneva, Switzerland; 4 Department of Infectious Disease Epidemiology, School of Public Health, MRC Centre for Global Infectious Disease Analysis, Imperial College London, Medical School Building, St Mary's Campus, Norfolk Place London W2 1PG, UK; 5 Department of Infectious Disease Epidemiology, London School of Hygiene and Tropical Medicine, Keppel St, London WC1E 7HT, UK; 6 Centre for Mathematical Modelling of Infectious Diseases, London School of Hygiene and Tropical Medicine, Keppel St, London WC1E 7HT, UK; 7 Clinical Research Department, Faculty of Infectious and Tropical Diseases, London School of Hygiene and Tropical Medicine, Keppel St, London WC1E 7HT, UK; 8 UK Public Health Rapid Support Team, London School of Hygiene and Tropical Medicine, Keppel St, London WC1E 7HT, UK; 9 Public Health England, Wellington House, 133–155 Waterloo Road, London SE1 8UG, UK; 10 Centre for Health Informatics, Computing and Statistics (CHICAS), Lancaster Medical School, Lancaster University, Lancaster LA1 4YW, UK; 11 School of Life Sciences, University of Sussex, Sussex House, Brighton BN1 9RH, UK

**Keywords:** epidemics, infectious, methods, tools, pipeline, software

## Abstract

Despite continued efforts to improve health systems worldwide, emerging pathogen epidemics remain a major public health concern. Effective response to such outbreaks relies on timely intervention, ideally informed by all available sources of data. The collection, visualization and analysis of outbreak data are becoming increasingly complex, owing to the diversity in types of data, questions and available methods to address them. Recent advances have led to the rise of *outbreak analytics*, an emerging data science focused on the technological and methodological aspects of the outbreak data pipeline, from collection to analysis, modelling and reporting to inform outbreak response. In this article, we assess the current state of the field. After laying out the context of outbreak response, we critically review the most common analytics components, their inter-dependencies, data requirements and the type of information they can provide to inform operations in real time. We discuss some challenges and opportunities and conclude on the potential role of outbreak analytics for improving our understanding of, and response to outbreaks of emerging pathogens.

This article is part of the theme issue ‘Modelling infectious disease outbreaks in humans, animals and plants: epidemic forecasting and control‘. This theme issue is linked with the earlier issue ‘Modelling infectious disease outbreaks in humans, animals and plants: approaches and important themes’.

## 1. Introduction

Emerging infectious diseases are a constant threat to public health worldwide. In the past decade, several major outbreaks, such as the 2009 influenza pandemic [1], the Middle-East Respiratory Syndrome coronavirus (MERS-CoV) [2–4], the emergence of Zika [5,6] and the West African Ebola virus disease (EVD) outbreak [7,8], have been potent reminders of the need for robust surveillance systems and timely responses to nascent epidemics [9]. The West African EVD outbreak, by far the largest such epidemic in recorded history, in particular, had a strong impact on global health security and public health policy and practice [7,8,10]. It highlighted the difficulties of maintaining situational awareness in the absence of standards for surveillance, data collection and analysis, as well as the challenges of mounting and sustaining a large-scale international response [7,8,11,12]. Despite the lessons learnt [9,13,14], the recent (2018) EVD outbreaks in Democratic Republic of the Congo [15,16] are a stark reminder that a large number of these challenges remain.

An important feature of the modern response to epidemics is the increasing focus on exploiting all available data to inform the response in real time and allow evidence-based decision making [3,4,7,8,13,17]. Using data for improving situational awareness is complex, involving a range of inter-connected tasks and skills from point-of-care data collection to the generation of informative situational reports (sitreps). The science underpinning these data pipelines involves a wide range of approaches, including database design and mobile technology [18,19], frequentist statistics and maximum-likelihood estimation [7], interactive data visualization [20,21], geostatistics [22–24], graph theory [20,25,26], Bayesian statistics [8,27,28], mathematical modelling [29–31], genetic analyses [32–36] and evidence synthesis approaches [37]. This accretion of heterogeneous disciplines, which may be best summarized as ‘outbreak analytics’, forms an emerging domain of data science: an ‘interdisciplinary field that uses scientific methods, processes, algorithms and systems to extract knowledge and insights from data in various forms' [38], dedicated to informing outbreak response. *Outbreak analytics* sits at the crossroads of public health planning, field epidemiology, methodological development and information technologies, opening up exciting opportunities for specialists in these fields to work together to meet the needs for an epidemic response.

In this article, we outline this developing research field and review the current state of outbreak analytics. In particular, we focus on how different analysis components interact within functional workflows, and how each component can be used to inform different stages of an outbreak response. We discuss key challenges and opportunities associated with the deployment of efficient, reliable and informative data analysis pipelines and their potential impact.

## 2. The outbreak response context

### (a) The different phases of an outbreak response

**Figure 1.**
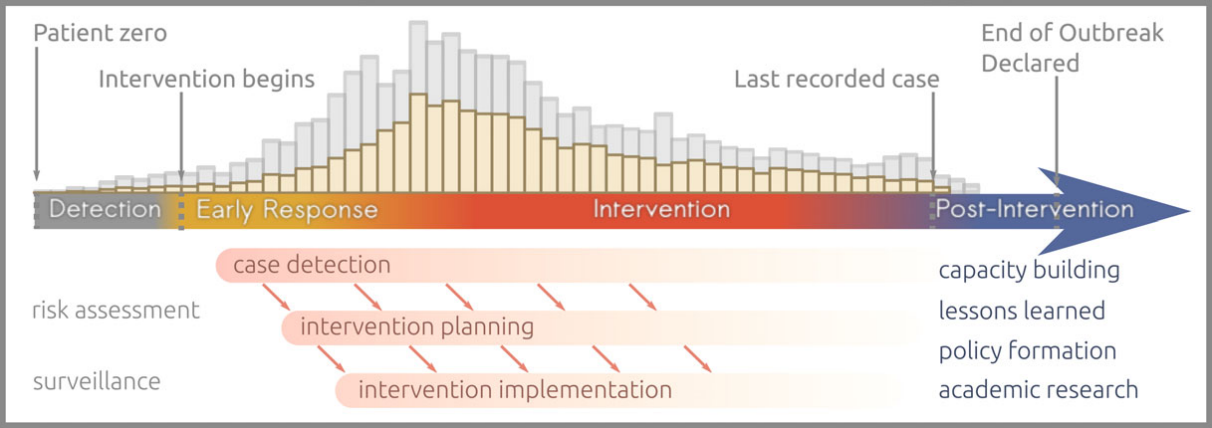
Successive phases of an outbreak response. The histogram along the top represents reported (yellow) and unreported (grey) incidence.

The focus of the public health response shifts during the course of an epidemic or outbreak, and so do the analytics. We identify four main stages (figure 1). The *detection* stage starts with the first case and ends with the first intervention activities (e.g. patient isolation, contact tracing, vaccination) and involves surveillance systems and mostly qualitative risk assessments. Next, the *early response* is the initial part of the intervention during which the first simple analytics can take place, essentially centred around estimating transmissibility. This blends into the *intervention* stage, where more complex analytics may be involved to inform planning (e.g. vaccination strategies), which ends once the last reported case has recovered or died. The *post-intervention* stage is for lessons to be learned, for improving preparedness for the next epidemic and for training and capacity building [39].

### (b) Questions during and after the intervention

During the early response, efforts are dedicated to estimating the likely impact of the outbreak and anticipating the nature, scale and timing of resources needed [7,13,15]. Theoretically, different factors including not only the total number of cases and fatalities but also the morbidity and overall impact on quality of life, as well as societal and economic impact, should ideally be taken into account when attempting to predict disease burden [40–43]. Generally, as the demographic and morbidity data needed by composite measures of health-adjusted life years [40] are lacking in outbreak response contexts, efforts tend to focus on other proxies of impact: assessing transmissibility, predicting future case incidence and associated mortality and investigating risk factors [1,3,7,15].

Analytical needs to diversify as the intervention progresses. While investigations of transmissibility, mortality and risk factors remain key throughout [8], new questions may arise to inform the implementation of control and mitigation measures. These may focus on predicting the impact of potential measures including testing (e.g. ‘Could a rapid test help reduce incidence?’ [29]), vaccine development (e.g. ‘Could a candidate vaccine be evaluated in this outbreak?’ [44,45]), vaccination campaigns (e.g. ‘Which is the optimal vaccination strategy?' [46,47]) or travel restrictions (e.g. ‘Should international travel be restricted?’ [48]), or on estimating the impact of current measures such as improvements in access to care (e.g. ‘Has the delay between symptom onset and hospitalization been reduced?’ [14,15]). As case incidence reduces, statistical modelling can also be useful for assessing or predicting the end of an outbreak [49–51]. At the field operational level, outbreak response analytics may be best focused on informing and monitoring core surveillance activities and performance indicators, such as contact tracing [11], through the use of tools for contact data visualization [52], mapping [53,54] and on analysis pipelines integrating mobile data collection tools [18,19,55,56] with automated reporting systems [57–59]. Finally, the post-intervention phase lends itself to retrospective studies, which can assess further the impact of interventions [60], tease apart finer processes driving the epidemic dynamics such as contact patterns [12,61], study risk factors [54,62], identify avenues for fortifying surveillance [13,36,63] and evaluate, improve and develop modelling techniques [28,64,65].

### (c) What are outbreak data?

The term ‘*outbreak data*’ encompasses different types of information, of which we first distinguish ‘*case data*’ from ‘*background data*’. *Case data* include the description of reported cases gathered in *linelists*, i.e. flat files where each row is a case and each column a recorded variable (e.g. dates of onset and admission, gender, age, location), thereby fulfilling the definition of ‘tidy data’ in the data science community [66]. Case data also include exposure and contact tracing data, either stored within a linelist or in separate files, pathogen whole genome sequencing (WGS) and data pertaining to outbreak investigations (e.g. case–control and cohort study data). *Background data* document the underlying characteristics of the affected populations. This includes demographic information (e.g. maps of population densities, age stratification, mixing patterns), movement data (e.g. borders, traveller flows, migration), health infrastructure (e.g. healthcare facilities, drug stockpiles) and epidemiological data themselves (e.g. levels of pre-existing immunity). A final type of data we consider here is ‘*intervention data*’, which refers to information on decisions made and efforts deployed as part of the intervention, such as vaccination coverage, the extent of active case finding or potential changes in the epidemiological case definition. An in-depth discussion of data needs in outbreaks can be found in Cori *et al*. [13].

## 3. Outbreak analytics

### (a) An overview of the outbreak analytics toolbox

**Figure 2.**
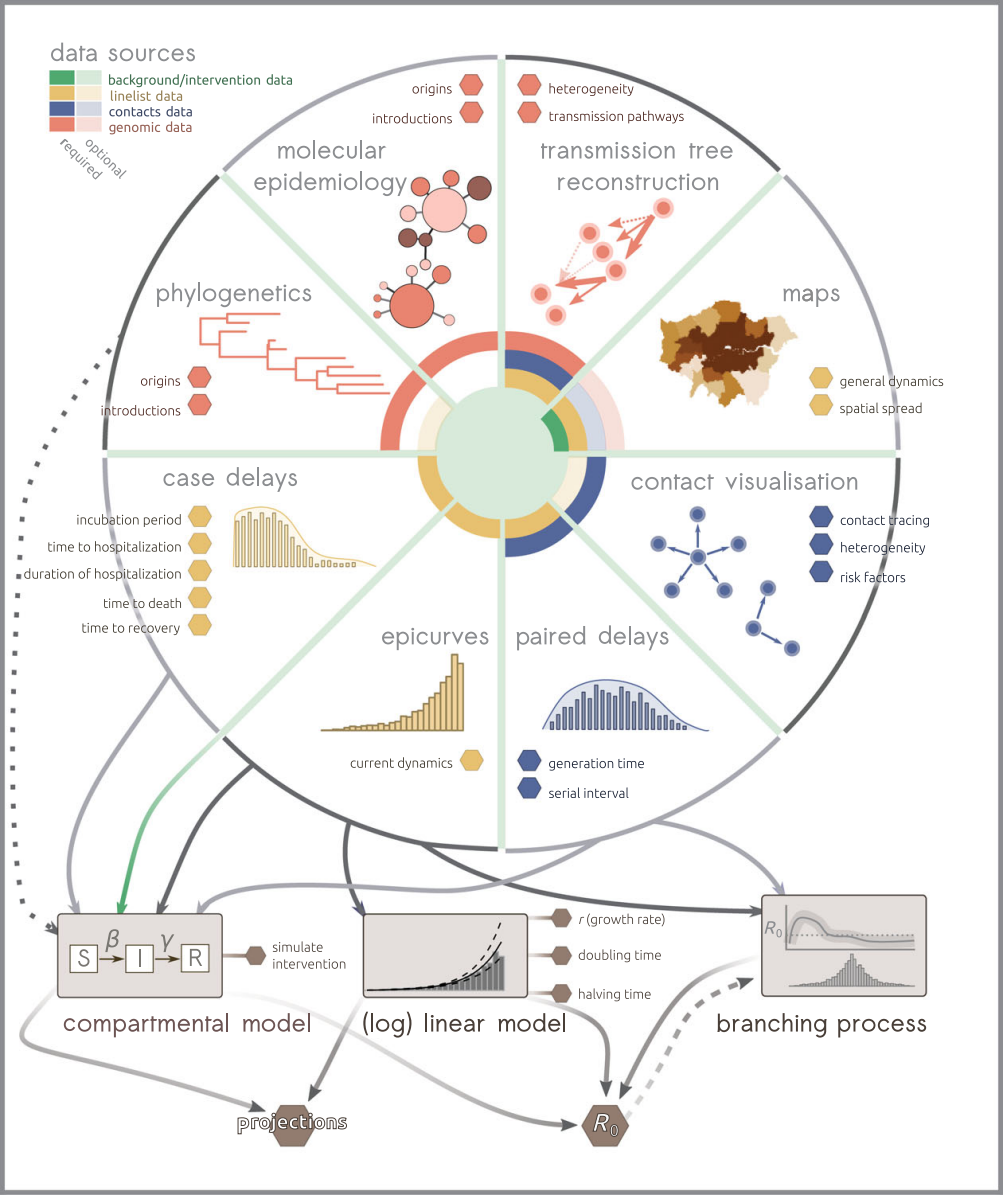
Example of outbreak analytics workflow. This schematic represents eight general analyses that can be performed from outbreak data. Outputs containing actionable information for the operations are represented as hexagons. Data needed for each analysis are represented as a different colour in the center, using plain and light shading for mandatory and optional data, respectively. (Online version in colour.)

We use the term ‘*outbreak analytics*’ to refer to the variety of tools and methods used to collect, curate, visualize, analyse, model and report on outbreak data. These tools and their inter-dependencies are summarized in an exemplary workflow represented in figure 2, derived from analyses pipelines used during recent epidemics of pandemic influenza [1], MERS-CoV [4] and EVD [7,8,17]. Note that workflows may vary substantially in other epidemic contexts. For instance, analyses of food-borne outbreaks may focus on traceback data [67–69], while vector-borne disease analysis may focus heavily on modelling the vector's ecological niche [70,71].

### (b) Tools for the collection of epidemiological data

Tools for data capture have become a focus of much discussion in recent years as those involved in outbreak response seek to make use of important technological advances including mobile data collection, cloud computing and built-in automated data analyses and reporting. In resource-limited settings, in particular, epidemiological data are still often collected with pen and paper, the advantages of which are familiarity, simplicity, low cost and reliability where access to Internet and power sources may be limited. However, there are some downsides to using paper as a data management tool, becoming increasingly important with larger outbreaks, as any system for the printing and distribution, collection and storage and digitization of forms becomes overwhelmed. Additionally, two-stage processes involving transcription of data from forms typically introduces additional data entry errors [72–75] and substantial delays from data capture to analysis [72].

Electronic data collection (EDC) is becoming increasingly popular [18,19,55,56]. These tools make use of widely available, low-cost hardware (e.g. smartphones and tablets) [76] that can, when appropriately configured, consume little power and collect data offline, making them suitable for use in resource-poor settings. Some of those may be part of existing surveillance systems or be deployed instead for specific enhanced surveillance and response activities during an outbreak. EDC platforms can also enhance data quality through the use of restriction rules and logical checks, and enforce reporting (even when there are zero cases) and entry of essential variables [72,76]. EDC can decrease the delay between data collection, centralization and analysis, which is critical for data-driven responses. Time can be saved through ‘form logic’ (e.g. automatically skipping sections of a survey not relevant to a participant), while real-time, automated centralization, data analysis and reporting can be directly built into the platform. In addition, mobile-based EDC enables the collection of other types of data including GPS coordinates, photographs, barcode (useful to link case data and clinical specimens) and even aiding diagnostics by directly interfacing with point-of-care diagnostic devices [77–79].

Maintaining confidentiality and privacy is a legitimate concern whenever data concerning human subjects are collected. While EDC systems provide opportunities for unauthorized interception and access to such information, many systems support end-to-end encryption during data transfer [80], although few provide additional security through encryption at the level of data entry.

### (c) Descriptive analyses

The first, and arguably one of the most important steps in data analysis is exploration, where visualization plays a central role, completed with informative summary statistics [81,82]. The first type of graphics needed for rapid assessment of ongoing dynamics is the epidemic curve (epicurve), which shows case incidence time series as a histogram of new onset dates for a given time interval [83–85]. Cumulative case counts, sometimes used in the absence of a raw linelist, are best avoided in epicurves, as they tend to obscure ongoing dynamics and create statistical dependencies in data points that will result in biases and lead to under-estimating uncertainty in downstream modelling [86].

Maps have been at the core of infectious disease epidemiology from a very early stage [87]. Nowadays, they are typically used to visualize the distribution of disease [88], for representing the ‘ecological niche’ of infectious diseases at large scales [23,24,89] and for assessing the spatial dynamics of an outbreak and strategizing interventions [7,8]. Providers of free and crowd-sourced [90] geographical data like the Humanitarian Open Street Maps Team (Humanitarian OpenStreetMap Team Home; see https://www.hotosm.org/ (accessed 26 September 2018)), the Missing Maps project (MissingMaps; see https://www.missingmaps.org/ (accessed 26 September 2018)), healthsites.io (see https://healthsites.io/ (accessed 26 September 2018)) and the Radiant Earth Foundation (Radiant Earth Foundation – Earth imagery for impact; see https://www.radiant.earth (accessed 18 November 2018)) provide layers of spatial data that include information on the location of households and health facilities, among other determinants. Several tools including SaTScan and ClusterSeer are routinely applied to surveillance system data for automated outbreak detection and the evaluation of clustering of disease by time and space [91]. Other examples of freely available mapping tools that can help track the spread of infectious diseases include the Spatial Epidemiology of Viral Haemorrhagic Fevers (VHF) disease visualization (see http://www.healthdata.org/datavisualization/spatial-epidemiology-viralhemorrhagic-fevers; accessed 19 September 2018), which maps risks of emergence and spread of VHF diseases, Nextstrain [92] and Microreact [93], which focus on mapping pathogen evolution and epidemic spread, and HealthMap [94], which provides resources for the rapid detection of outbreaks. Geographical locations of reported cases can also be useful for informing more complex modelling approaches [95].

In epidemics driven by person-to-person transmission, a last essential source of data is contact data [20], which includes data on case *exposure* [12] as well as *contact tracing,* where appropriate [11,63]. Exposure data document transmission pairs, which can yield precious insights into ‘paired delays’ (figure 2) including the serial interval (time between onsets of a case and their infector) or the generation time (time between the dates of infections of a case and their infector) [7,8], which are in turn useful for estimating transmissibility [27,28,96,97]. Exposure data can also be used to investigate the occurrence and determinants of super-spreading events [12] and help identify introduction events in the case of zoonotic diseases [98]. Contact tracing, through the early detection of new cases and their subsequent isolation and treatment, plays a central role in reducing onward transmission and therefore containing outbreaks [11,63,99], while additionally providing potential information on risk factors [7,11].

Summary statistics are a useful complement to data visualization in the exploratory phase of data analysis. Some metrics, such as transmissibility, require the use of statistical or mathematical models in order to be estimated (see §3d below) and are therefore not readily available as descriptive tools. Other useful statistics can be readily computed from linelists, including different demographic indicators of the reported cases (e.g. gender, age, occupation [7,100,101]), case fatality ratios (the proportion of cases who died of the infection) or case delays such as the times to hospitalization, recovery or death, reported as a whole [1,7,8] or stratified by groups [100,101]. The incubation period (time from infection to symptom onset) is another important delay for informing the intervention (e.g. to define the duration of contact tracing or declare the end of an outbreak), but can be harder to derive as it requires data on case exposure as well. Note that in the case of delays, these are best analysed by characterising the full distribution (e.g. by fitting to an appropriate probability distribution such as discretized Gamma [7]) rather than reported as a single central value [7,8,102,103].

### (d) Quantifying transmissibility

The ‘transmissibility’ of a disease is here used to refer to the rate at which new cases arise in the population, resulting either in epidemic growth or decline [1,3,27,28]. Rather than an intrinsic property of a specific disease, transmissibility thus defined quantifies the propagation of a pathogen in a given epidemic setting and is impacted by multiple factors including population demographics, mixing and levels pre-existing immunity. Importantly, estimates of transmissibility reported in the literature will typically be biased towards higher values, as subcritical outbreaks are by definition less likely to be detected. Several metrics of transmissibility can be used depending on the type of data available and can be estimated using different approaches.

A first measure of transmissibility is the *growth rate* (*r*), which is estimated from a simple model where case incidence is either exponentially growing (*r* > 0) or declining (*r* < 0). Typically, *r* is estimated directly from epicurves (figure 2) using a log-linear model, where *r* is defined as the slope of a linear regression on log-transformed incidence [104,105]. Besides its simplicity and its computational efficiency, this approach has the benefits of being embedded in the linear modelling framework, thereby allowing one to measure the uncertainty associated with a given estimate of *r*, to test for differences in growth rates, e.g. between different locations, and to derive short-term incidence predictions. Moreover, the growth rate can also be used to estimate the doubling and halving times of the epidemic, i.e. the time during which incidence doubles (respectively is halved), as alternative metrics of transmissibility [103]. Unfortunately, the log-linear model can only fit exponentially growing or decaying outbreaks, which may not always be appropriate in the presence of complex spatial or age structure, or owing to changes in reporting, transmissibility or proportion of susceptible individuals over time. Besides, it cannot readily accommodate time periods with no cases, so that its applicability may in practice be restricted.

While *r* quantifies the *speed* at which a disease spreads, it does not contain information on the *level* of the intervention that is necessary to control a disease [106]. This is better characterized by the *reproduction number* (here generically noted ‘*R*’), which measures the average number of secondary cases caused by each primary case. Researchers typically distinguish the basic reproduction number (*R*_0_ [104]), which applies in a large, fully susceptible population, without any control measures, from the effective reproduction number (*R*_eff_), which is the number of secondary cases after accounting for behavioural changes, interventions and declines in susceptibility [96]. The current reproduction number determines the dynamics of the epidemic in the near future, with values greater than 1 predicting an increase in cases, and values less than 1 predicting control [104]. The value of *R* can also be used to calculate the fraction of the population that needs to be immunized (typically through vaccination) in order to contain an outbreak [104].

Different methodological approaches have been developed to estimate the reproduction number. *R* can be approximated using estimates of the growth rate *r* combined with knowledge of the generation time distribution [97]. *R* can also be derived from compartmental models [104,107]. The formula will depend on the type of model used, but such estimation will usually require that different rates (e.g. rates of infection, recovery, death) are either known or estimated by fitting the model to data [104,107]. Real-world complexities can be incorporated into this approach; however, fitting such models can be challenging and may require computationally intensive algorithms such as data augmentation, approximate bayesian computation, or particle filters [108]. Compartmental models also require assumptions about the total population size and the proportion of the population at risk, which may be difficult to estimate in an outbreak. As an alternative, branching process models can be used to estimate *R* directly from incidence data [27,28,96,109]. This requires a pre-specified distribution of the generation time, or of the serial interval, although recent developments suggest that in some cases, the generation time distribution itself can also be simultaneously estimated [4]. Branching process models are usually much simpler to fit to data than their compartmental counterparts, which facilitates their use in real time [27].

Beyond the mere estimation of transmissibility, it is often essential to forecast future incidence for advocacy and planning purposes, e.g. to compare different interventions and epidemic scenarios [7,8,15,30]. A variety of mathematical and statistical models, including those reviewed here for estimating transmissibility, can also be used for short-term incidence forecasting [65]. Despite the growing body of research focusing on predicting incidence during epidemics [65,110], there are currently no gold standards and the relative performances of forecasting methods largely remain to be assessed. Methods that have been developed and applied in other fields to rigorously assess not just the accuracy of forecasts but also how well models quantify the inherent uncertainty in making predictions, are only rarely applied in infectious disease epidemiology [111,112]. Whether it is to estimate *R* or predict future incidence, the most appropriate method ultimately depends on the particular epidemiological setting, existing knowledge of the transmission dynamics and data availability. Branching process models, for example, can be used for a quick estimate of the current value of *R* from the recent trend in case numbers and, by extrapolating this forward, of expected case numbers in the near future [27,28,96]. Mechanistic or simulation models, on the other hand, aim to include a more explicit representation of the different factors that might influence transmission. They can be a more natural choice for assessing the expected impact of possible interventions, but they usually require careful parametrization and often intensive computation [29,30,45,113], both of which can be challenging early in an outbreak when data are scarce and rapid turnaround crucial.

### (e) Analytical epidemiological techniques

Analytical epidemiological studies use data to better describe outbreaks and populations at risk and inform real-time and subsequent response efforts. Typically, these are conducted during the intervention and post-intervention phases of an outbreak response (figure 1). They include observational designs such as retrospective cohort and case–control studies to identify risk factors and quantify associations between potential causes and their outcomes (typically, the disease in question), and experimental designs, such as randomized-control studies used to estimate the impact of interventions such as vaccination and treatments [114]. These studies reside outside of the normal scope of outbreak response activities, being inserted *ad hoc* as functions that are not necessarily routine response activities such as strengthening surveillance. In the case of observational epidemiological studies, data on exposures and outcomes are required, permitting estimations of the increased risk of disease among people exposed to risk factors of interest [54,62,115,116]. In the case of experimental epidemiology, data on outcomes of interest are collected to permit estimations of heterogeneity among groups (e.g. in the presence/absence of intervention).

The usefulness of such studies in informing outbreak response is highly context-dependent. Observational studies may be undertaken early on in the intervention phase to help identify ongoing infection sources of environmental, food-borne or water-borne nature [117] and to stop the outbreak at its source. In longer-running outbreaks, they can provide insights into opportunities for control [53,115,118] and inform global policy decisions that relate to outbreak response [119]. However, the time and expertise needed to prepare and implement these studies may preclude their application in the midst of an ongoing outbreak, so that the cost and benefits of such an undertaking need to be carefully weighed in emergency settings.

### (f) Genetic analyses

Whole genome sequencing of pathogens is increasingly affordable and reliable, and therefore more frequent in outbreak investigations [1,120–126]. As technology is making real-time sequencing in the field a developing standard in the coming years [127,128], genetic analysis will likely carve out its own space in the outbreak analytics toolkit.

Different methods can be used to extract information from pathogen WGS. In bacterial genomics, molecular epidemiology methods have been used extensively for defining strains of related isolates [32,129], which can be used to infer various features of the pathogens sampled such as their origins, antimicrobial resistance profiles, virulence or antigenic characteristics [130–132]. These methods usually exploit only a fraction of the information contained within pathogens' genomes, as they rely on genetic variation in a limited number of housekeeping genes [32,129]. While these methods will likely remain useful in years to come, substantially more information can be extracted by using WGS to reconstruct phylogenetic trees, which represent the evolutionary history of the sampled isolates, assuming the absence of selection or horizontal gene transfers [133]. Different types of phylogenetic reconstruction methods can be used, including fast, scalable distance-based methods [134] or more computer-intensive approaches using a maximum-likelihood [135,136] or the Bayesian framework [33,137]. Phylogenies can be used to assess the origins of a set of pathogens [138], patterns of geographical spread [125], host species jumps [139,140], past fluctuations in the pathogen population sizes [141] and even, in some cases, the reproduction number [1]. Importantly, there is a growing tendency to analyse phylogenetic trees in the broader context of other epidemiological data (mainly geographical locations until now), which is facilitated by user-friendly Web applications [92,93].

A further step towards integrating WGS alongside epidemiological data is the reconstruction of transmission trees (who infects whom) using evidence synthesis approaches. This methodological field has been growing fast over the past decade [25,142–148], but most applications of these methods remain within academia and their usefulness in the field in an outbreak response context needs to be critically assessed. A potential benefit of accurately reconstructing transmission trees lies in the identification of multiple introductions, the quantification of the proportion of unreported cases and the detection of heterogeneities in individual transmissibility [145]. Unfortunately, the reconstruction of transmission trees is a difficult and computationally intensive problem. First, most diseases do not accumulate sufficient genetic diversity during the course of an outbreak to allow the accurate reconstruction of transmission chains, so that multiple data sources need to be used [35], making these methods more data-demanding than most other approaches in outbreak analytics (figure 2). In addition, the complex nature of the problem requires the use of Bayesian methods for model fitting, making these approaches difficult to interpret by non-experts [145,146,148].

## 4. Discussion

In this article, we reviewed methodological and technological resources forming the basis of outbreak analytics, an emerging data science for informing outbreak response. Outbreak analytics is embedded within a broader public health information context that starts with disease surveillance systems, followed by risk assessment and management, the epidemiological response itself, and finishes with the production of actionable information for decision making. Part of the challenge that this new field will face in the coming years pertains to the seamless integration of data analytics pipelines within existing workflows. As responders can allocate only limited time to data analysis, analytics resources should produce simple, interpretable results, highlighting the most pressing issues that need addressing and monitoring all relevant indicators to inform the response.

Outbreak analytics and resulting outputs are central to the surveillance pillar of any outbreak response, yet resources and capacities to ensure data availability and quality are often limited owing to operational constraints [16]. Priorities in terms of data needs should be defined by what actionable information it may give access to through the available analytics pipelines [13]. In this respect, we foresee that typical linelist data such as dates of events (e.g. onset, reporting, hospitalization, discharge), age, gender, disease outcome, geographical locations and exposure data will fulfil most needs, while other data such as WGS may only be useful for specific diseases and contexts [34,35]. Intervention data are rarely collected but should be given more consideration, as they are key to assessing the impact and effectiveness of control measures, both during and after the operations. Similarly, data on the fraction of cases reported (and its variations through time), as well as behavioural changes (e.g. care-seeking behaviour) in the affected populations, can be very important sources of information for modelling [149].

Fortunately, what we called ‘background data’ in this article can be gathered and shared outside of the epidemic context. Besides maps, population census, sero-surveys or genetic databanks, data on the natural histories of diseases derived from past epidemics, such as key delay distributions and transmissibility, can form a useful substitute to real-time estimates, especially in the early stages of outbreaks when such data may be lacking. While crowd-sourced initiatives are promising and have been used successfully in low resource settings [90], more efforts are needed to collate and curate open data sources, assess their quality and make them widely available to the community. We argue that international public health agencies and non-governmental agencies should play a central role in orchestrating such background data preparedness.

Outbreak analytics is a developing field, and as such, there remain many gaps in terms of data collection, analysis and reporting tools. Some methodological challenges persist, such as better characterising forecasting methods [28,64,65], including spatial information and population flows into existing transmission models [95], and improving the integration of different types of data for reconstructing transmission trees [35]. In order to ensure transparent methods and availability to analysts in any setting, the implementation must be as freely available, open-source software. Among other popular programming languages, such as Python, Java, or Julia, the R software [150] arguably offers the largest collection of free tools for data analysis and reporting, and an increasing number of packages for infectious disease epidemiology [20,21,27,84,145] may form a solid starting point for the development of a comprehensive, robust and transparent toolkit for the analysis of epidemic data [151]. Importantly, the use of a common platform for the development and use of outbreak analytics tools will also likely contribute to standardizing data practices, including collection, sharing and analysis.

A final point relates to the use and dissemination of these new resources: how can outbreak analytics best help improve public health? As noted by Bausch & Clougherty [39], *health science should not be an entity unto itself, but a means to an end*. Insofar as it can help field epidemiologists collect, visualize and analyse data, and subsequently provide decision-makers with actionable information, outbreak analytics will likely occupy an increasing space in field epidemiology over the years to come. We foresee that the dissemination of free training resources [152], the modernization of field epidemiology training programmes and the deployment of applied data scientists to the field with a sustained capacity building in resource-poor and vulnerable countries will be instrumental in shaping the future of this emerging field of health science.

## Data accessibility

This article has no additional data.
